# Supplementary material for: Optical Investigation of Action Potential and Calcium Handling Maturation of hiPSC-Cardiomyocytes on Biomimetic Substrates
Source: Int J Mol Sci. 2019 Aug 3;20(15):3799. doi: 10.3390/ijms20153799 (PMC6695920; doi:10.3390/ijms20153799)
Supplement: Supplementary file 1 [file ijms-20-03799-s001.zip › IJMS-558294-supplementary material.pdf]

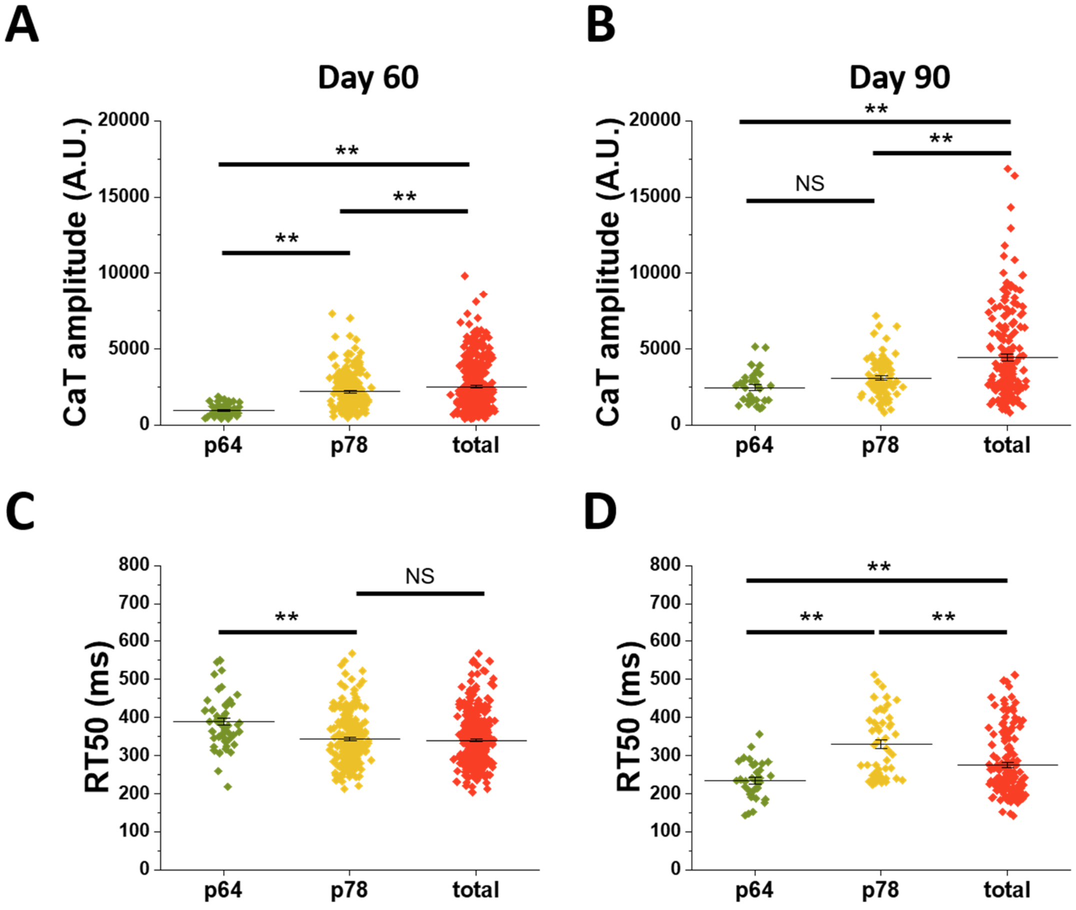

Fig. S1. Evaluation of intervariability of CaT parameters within differentiation batches of hiPSC-CMs. Individual differentiation runs (from individual passages, p) were plotted to compare the distribution and against the average of total differentiations of hiPSC-CM (total) for CaT amplitude at both (A-B) day 60 and day 90 together with (C-D) CaT decay (RT50, ms) distribution of single hiPSC-CMs. Standard deviation was used to analyze distribution comparison. One-way analysis of variance (ANOVA) with a Tukey post-hoc test with statistical significance set at $p<0.05$* and $p<0.01$**; NS not significant.

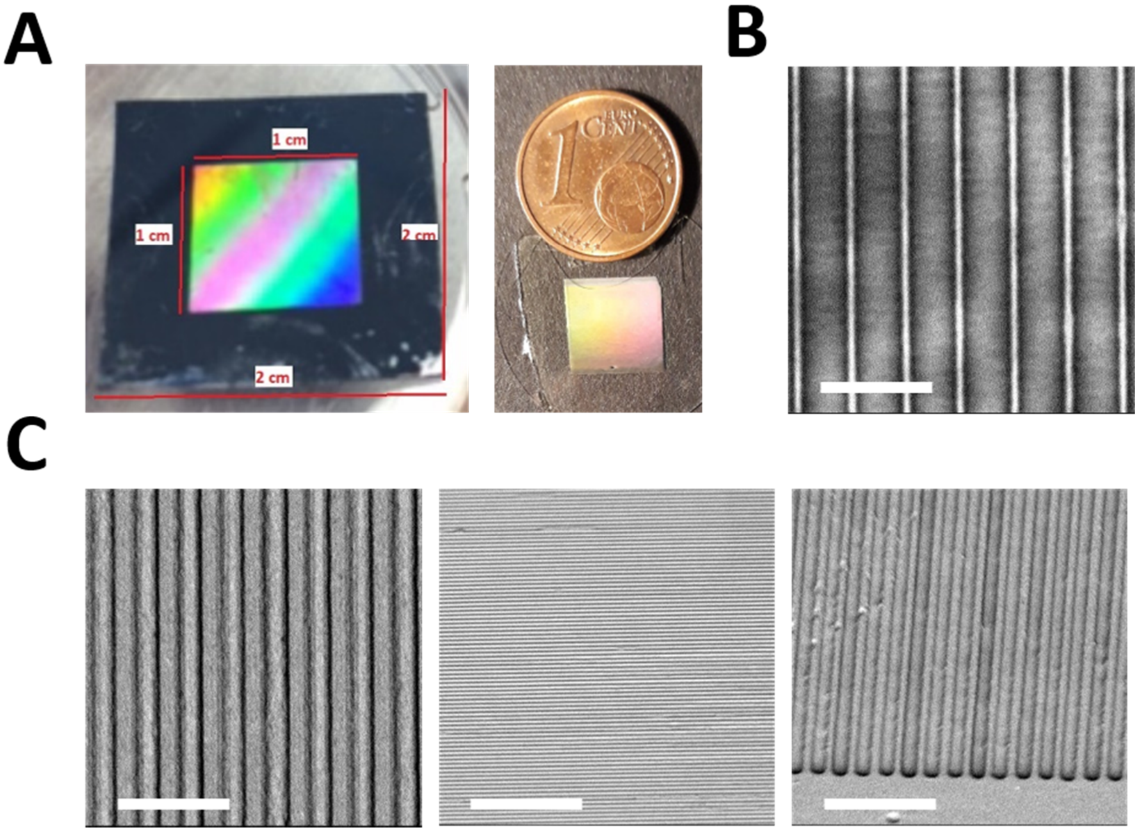

Fig. S2. Fabrication of hydrogel-based micropatterned surfaces. (A) Example of master used for the preparation of the PDMS mold employed in the litographic patterning of polyethilene-glycol (PEG) hydrogel. (B) SEM image of a silicon master with micro-grooves and ridges. (C) SEM images of the PEG hydorgel. Scale bars equal to 10μm.

Clinical / Demographic Data	
Gender	Female 4/10 (40%)
Age at surgery	59 ± 6 yrs
Presence of known cardiomyopathies	0/10 (0%)
NYHA Class I	8/10 (80%)
NYHA Class II	2/10 (20%)
Atrial Fibrillation	0/10 (0%)
**Medical Treatment**	
Beta blockers	6/10 (60%)
Diuretics/ACE-Inhibitors	3/10 (30%)
**Echo features**	
Maximal LV wall thickness	12 ± 2 mm
LA end-systolic volume	82 ± 16 mL
Ejection fraction	60 ± 5 %
LVOT gradient >30mmHg	0 /10 (0%)
Bulging septum	10/10 (100%)
**Reason for Surgery**	
Aortic valve steno-insufficiency	6/10 (60%)
Mitral valve prolapse	2/10 (20%)
Mitral valve steno-insufficiency	1/10 (10%)
Ascending aorta dilatation	1/10 (10%)

Table S1. Clinical features of control patients

The table includes pre-operative data from the 10 patients from whom myocardial tissue specimens were obtained and studied. Data are indicated as Mean ± SD.

LV = left ventricle

LA = left atrium

LVOT = left ventricular outflow trait

hiPSC-CMs	Pacing rate	day 60	day 75	day 90	day 90 FSK	day 90 ISO	hAdult-CMs
APD50 (ms)	1Hz	193±40*	291±7	362±9		229±12	325±31
	2Hz	193±30*	259±5	248±5			
CaT amplitude (A.U.)	1Hz	2515±93	2036±73	4849±273	7470±479	5960±741	
	2Hz	1921±103	1087±50	2390±131			
CaT TTP (ms)	1Hz	234±3	181±5	188±6			95±9
	2Hz	180±3	134±3	157±3			
CaT RT50 (ms)	1Hz	340±4	315±7	277±7	260±6	226±5	255±26
	2Hz	238±4	223±5	202±4			
PR APD50 (ms)	2Hz		400±12	380±16			
PR RT50 (ms)	2Hz	483±11	387±25	334±18			
CaT PRP (A.U.)	2Hz	2335±161	1917±91	4380±205			

Table S2. Summary of action potential and calcium transient results from hiPSC- and hAdult-CMs. Time point experiments of dual recording are reported for hiPSC-CMs at 1Hz and 2Hz of pacing rate. Comparison with hAdult-CMs at 1Hz is reported. Asterisk (*) refers to patch clamp recordings. Data are reported as Mean±SEM.
